# “A hug of cuddles”

**DOI:** 10.1186/1824-7288-40-S2-A43

**Published:** 2014-10-09

**Authors:** Belvisi Rosaria

**Affiliations:** 1 Azienda Ospedaliera L. Sacco, Milan, Italy

## Venue

1st level Nursery, LUIGI SACCO HOSPITAL MILAN, 1250 births per year.

Late Preterm (LP) : 8%

## Target

Create procedures cannot break the creation of bonding: the process that contribute to form the bond between parents and children necessary to reach a mutual harmony .. In the LP this interactive dance where you learn to recognize, is less rigidly determined and you have to start it with a well-defined Care program.

## Participants

Late preterm infants, parents, and professionals who are trained with periodic refresher courses.

## Project

Smooth approach with new parents that are made immediately bring to their child.

Presentation of the structure, the incubator and equipment that monitor the baby.

Support in the first contact between parents and child.

Kangaroo therapy at different times and different way depending on the clinical condition of the child.

Ad hoc preparation of the room in order to involve all the senses of the newborn.

Involvement of parents in the care of the newborn LP: postural care, nest, wrapping, holding, hygiene care, serving meals, breast feeding, even if the baby is in the incubator.

Course with parents, aimed to autonomy.

Planned Hospital discharge

Massage Course

## Results

The impressions collected at discharge and beyond, demonstrate how this project is hugely important, not only for children but also for the well-being and balance of the newly couples, who often find themselves terrified and frightened by their child premature birth and they just need to be supported and accompanied in this path that leads to the creation of a unique and indissoluble bond like that with their children

